# Allele-Specific Knockdown of ALS-Associated Mutant TDP-43 in Neural Stem Cells Derived from Induced Pluripotent Stem Cells

**DOI:** 10.1371/journal.pone.0091269

**Published:** 2014-03-20

**Authors:** Agnes L. Nishimura, Carole Shum, Emma L. Scotter, Amr Abdelgany, Valentina Sardone, Jamie Wright, Youn-Bok Lee, Han-Jou Chen, Bilada Bilican, Monica Carrasco, Tom Maniatis, Siddharthan Chandran, Boris Rogelj, Jean-Marc Gallo, Christopher E. Shaw

**Affiliations:** 1 Department of Clinical Neuroscience, King's College London, London, United Kingdom; 2 Department of Public Health, Neuroscience, Experimental and Forensic Medicine, University of Pavia, Pavia, Italy; 3 MRC Centre for Regenerative Medicine and Centre for Neurodegeneration, University of Edinburgh, Edinburgh, United Kingdom; 4 Department of Biochemistry & Molecular Biophysics, Columbia University, New York, New York, United States of America; 5 Department of Biotechnology, Jozef Stefan Institute, Ljubljana, Slovenia; International Centre for Genetic Engineering and Biotechnology, Italy

## Abstract

TDP-43 is found in cytoplasmic inclusions in 95% of amyotrophic lateral sclerosis (ALS) and 60% of frontotemporal lobar degeneration (FTLD). Approximately 4% of familial ALS is caused by mutations in TDP-43. The majority of these mutations are found in the glycine-rich domain, including the variant M337V, which is one of the most common mutations in TDP-43. In order to investigate the use of allele-specific RNA interference (RNAi) as a potential therapeutic tool, we designed and screened a set of siRNAs that specifically target TDP-43^M337V^ mutation. Two siRNA specifically silenced the M337V mutation in HEK293T cells transfected with GFP-TDP-43^wt^ or GFP-TDP-43^M337V^ or TDP-43 C-terminal fragments counterparts. C-terminal TDP-43 transfected cells show an increase of cytosolic inclusions, which are decreased after allele-specific siRNA in M337V cells. We then investigated the effects of one of these allele-specific siRNAs in induced pluripotent stem cells (iPSCs) derived from an ALS patient carrying the M337V mutation. These lines showed a two-fold increase in cytosolic TDP-43 compared to the control. Following transfection with the allele-specific siRNA, cytosolic TDP-43 was reduced by 30% compared to cells transfected with a scrambled siRNA. We conclude that RNA interference can be used to selectively target the TDP-43^M337V^ allele in mammalian and patient cells, thus demonstrating the potential for using RNA interference as a therapeutic tool for ALS.

## Introduction

The TDP-43 proteinopathies are a group of diseases with overlapping clinicopathological features including amyotrophic lateral sclerosis (ALS) and frontotemporal lobar degeneration with TAR DNA-binding protein (TDP-43) inclusions (FTLD-TDP). The common hallmark of TDP-43 proteinopathies is the formation of phosphorylated, ubiquitinated and detergent-insoluble TDP-43 in the cytoplasm of motor neurons. In addition, cleavage of TDP-43 within the C-terminus produces lower molecular weight species of 35 and 25 kDa [1], [2].

TDP-43 is a DNA/RNA binding protein of 43 kDa mainly localized in the nucleus, and has been implicated in transcriptional repression, pre-mRNA splicing and translational regulation [3], [4], [5]. In cell culture, overexpressed full length TDP-43 is localized mainly in the nucleus, whereas C-terminal fragments containing RNA recognition motif 2 (RRM2) and the glycine-rich domain are localized both in the nucleus and cytoplasm with formation of ubiquitinated inclusions in the latter compartment [6], [7], [8], [9], . Recent studies have shown that the TDP-43 C-terminal fragments are prone to aggregation and may serve as a seed to facilitate aggregation of full-length TDP-43 [10].

Mutations in TDP-43 have been identified in familial and sporadic cases of ALS and FTLD-TDP, mainly in the C-terminal glycine-rich region, including the M337V mutation caused by an alteration of an adenine (A) to guanine (G) at position 1009 of *TARDBP* cDNA [3], [11], [12], [13], [14], [15], [16]. In a recent study using isogenic lines, mutant forms of TDP-43 were reported to be more stable than wild-type which was degraded two to four times faster than mutant TDP-43 [17]. Furthermore, mature motor neurons and neural stem cells (NSCs) derived from induced pluripotent stem cell (iPSC) lines from a patient carrying the M337V mutation showed higher levels of soluble and insoluble TDP-43 compared to controls. Given that overexpression of wild-type TDP-43 is toxic in a wide range of animal models [18], [19], the toxicity of mutant TDP-43 may be underpinned by its accumulation.

Regardless of the mechanism by which mutant TDP-43 exerts toxicity, selectively reducing expression of the mutant protein, while maintaining expression of wild-type TDP-43, is an attractive therapeutic strategy. One way to target the mutant allele in familial cases is using effective allele-specific small interference RNAs (siRNAs); an approach which has been already described in several autosomal dominant diseases such as Parkinson's disease [20], Alzheimer's Disease [21], [22] and Huntington's disease [23], [24], [25]. In ALS, silencing of a mutant superoxide dismutase 1 (SOD1) allele has been successfully achieved using siRNA and short hairpin RNA (shRNA) in cells and in animal models of ALS. It was shown that injection of shRNA delays ALS onset and extends survival in animal models [26], [27], [28], [29], [30].

To determine the effects of allele-specific siRNA as a potential therapeutic tool for familial ALS with mutation in TDP-43, we generated siRNAs specifically targeting the M337V mutant allele. These siRNAs were initially validated in HEK293T cells overexpressing full length GFP-TDP-43^wt^ or ^M337V^, and subsequently analysed in iPSC-derived cells.

Here we show for the first time that allele-specific siRNA decreases levels of mutant protein produced from the M337V allele, in NSCs derived from iPSCs from an ALS patient.

## Materials and Methods

All participants provided their written signed consent to donate their skin sample to derive iPSCs and this study was approved by the Ethics committee from the King's College Hospital, a National MREC (ethics ref. 10/S1103/10).

### Plasmids and siRNA

Full length TDP-43 cDNA was used as a PCR template to generate full length TDP-43, which was tagged with N-terminal green fluorescent protein (GFP) by subcloning into EGFP-C1 (Clontech Laboratories Inc, Mountain View, USA). The C-terminal GFP-TDP-43 constructs encompassing the amino acids 181–414 containing the RNA recognition motif 2 (RRM2) and the glycine-rich domain were amplified using the full length GFP-TDP-43 as a template and subcloned into EGFP-C1 vector. The mutant M337V counterparts were obtained by QuikChange site-directed mutagenesis of these constructs following manufacturer's instructions (Agilent technologies Inc, Santa Clara, USA). All plasmids were sequence-verified.

Human siRNAs were obtained from Invitrogen (Carlsbad, USA), which target the endogenous TDP-43 (named here as siTDP-43; Invitrogen Stealth). Scrambled non-targeting sequence was obtained from Invitrogen (named as sic; Invitrogen Stealth, low GC content).

**Figure 1:**
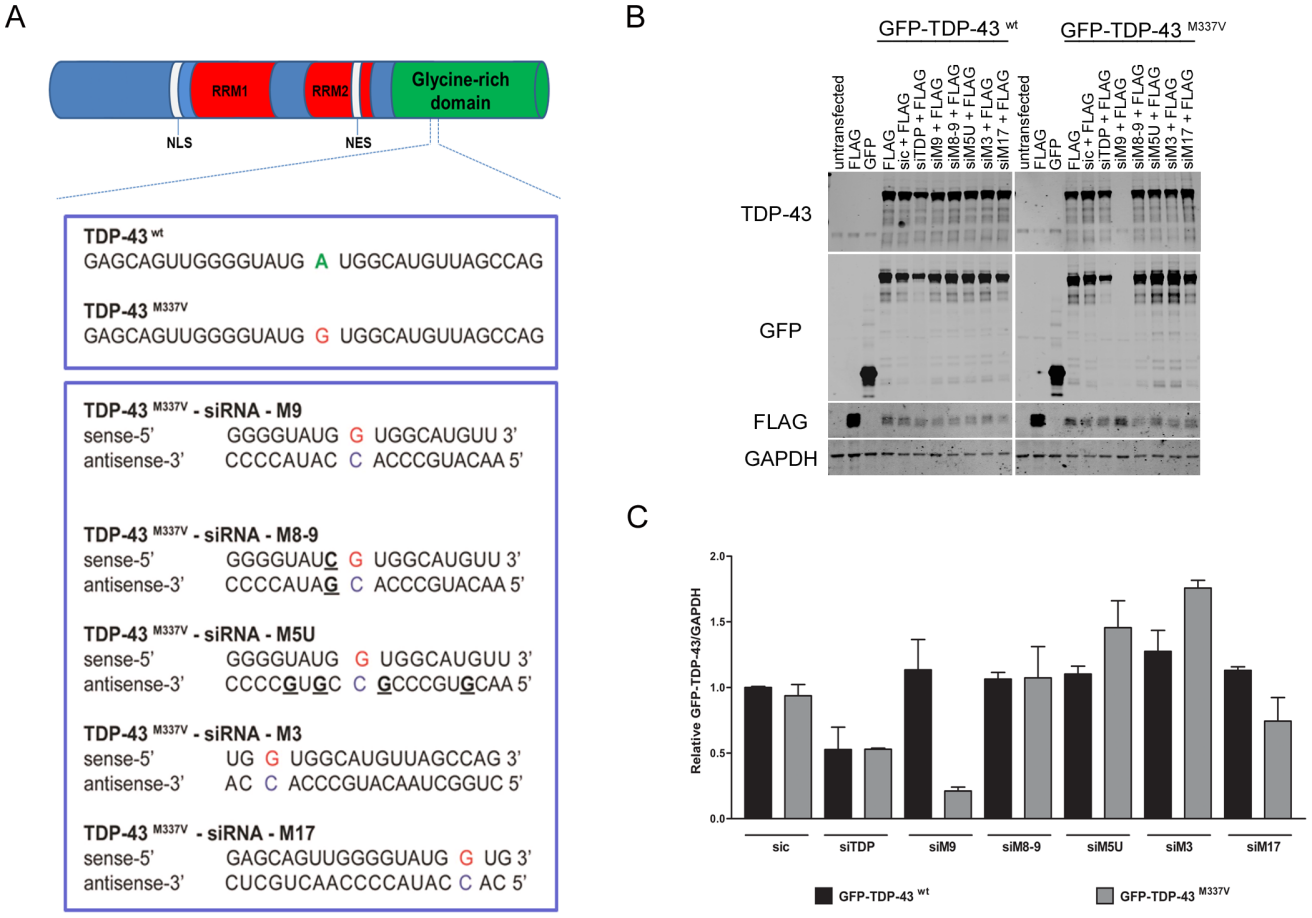
Allele-specific siRNAs targeting TDP-43^M337V^ mutant allele. **A.** Schematic representation of TDP-43 protein containing two RNA-recognition motifs (RRM1 and RRM2), a bipartite nuclear localization signal (NLS), a nuclear export signal (NES) and a glycine-rich domain in the carboxy-terminal. The M337V mutation localization is indicated. Five allele-specific siRNAs were designed to contain mismatches at positions 9 (M9), 3 (M3), or 17 (M17); double mismatches at positions 8 and 9 (M8-9) or multiple mismatches at positions 5, 7, 10 and 16 (M5U). **B.** Representative western blot image showing the effects of allele-specific siRNA on cells transfected with GFP-TDP-43^wt^ and GFP-TDP-43^M337V^. The allele-specific siM9 reduces the levels of GFP-TDP-43^M337V^ specifically whereas GFP-TDP-43^wt^ levels remain unchanged. FLAG-tagged protein was used as a control for transfection efficiency. **C.** Densitometry analysis of relative GFP-TDP-43 normalised to GAPDH. Mean from three independent experiments. Error bars represent standard error of the mean (SEM). (One way ANOVA, * P<0.05; *** P<0.001).

Five perfectly matched or single/multiple nucleotide mismatched siRNAs were designed to silence the M337V mutation with a single mismatch at position 9 (M9); 3 (M3); or 17 (M17); double mismatches at positions 8 and 9 (M89); and multiple mismatches at positions 5, 7, 10 and 16 (M5U) (Fig. 1). Allele-specific siRNAs were obtained from Eurogentec S.A. (Liège, Belgium) and from Invitrogen.

### Cell culture and transfection

All reagents used for cell culture were obtained from Invitrogen unless otherwise stated.

### HEK293T cells

HEK293T cells were cultured in DMEM high glucose with Glutamax, 100 U/mLpenicillin/100 µg/mL streptomycin and 10% foetal bovine serum. Cells were transfected with Lipofectamine siRNA Max following manufacturer's instructions. Initial allele-specific screening by western blot analysis was performed co-transfecting 800 ng of GFP-TDP-43^wt^ or GFP-TDP-43^M337V^ (full length) and 50 nM allele-specific siRNA. 5 nM of siRNA targeting endogenous TDP-43 (siTDP) and non-targeting siRNA (sic) were transfected into cells as experimental controls. Cells were analysed 48 hours post-transfection.

Stable, tetracycline-inducible clonal cell lines were generated in T-REx HEK293, cells (#R710-07, Invitrogen) using the T-REx system. Briefly, stable clonal lines transfected with pcDNA6/TR (constitutively expressed Tet-repressor) were transfected with pDEST30 HA-TDP-43 constructs using Lipofectamine 2000, selected using 600 µg/mL geneticin, and clonally isolated. Stable HEK293 lines were maintained as per HEK293T cells, but using Tet-free foetal bovine serum. All cells were maintained at 37°C, 5% CO_2_.

### Neural stem cells

Induced pluripotent stem cells were derived from one ALS patient carrying the M337V mutation and one normal control as previously characterized [31]. Cells were cultured feeder-free on matrigel-coated flasks with mTeSR1 (STEMCELL technologies, Vancouver, USA).

iPSCs were differentiated into neural stem cells (NSCs) as described elsewhere before differentiating into neurons [32]. Briefly, iPSCs were plated at 30–40% confluence in neural induction medium containing Advanced DMEM/F12:Neurobasal (1∶1), 100 U/mL penicillin/100 µg/mL streptomycin, 1% L-glutamine, 1× N2, 1× B27, 5 µg/mL BSA (Europa Bio-products, Cambridge, UK), 10 ng/mL hLIF (Millipore, Billerica, MA, USA), 3 µM CHIR99021 (BioVision technology Inc, San Francisco, USA), 2 µM SB431542 (Activin Inhibitor, Tocris Biosciences, Bristol, UK) and 0.1 µM Gamma Secretase Inhibitor XXI, Compound E (Merck Chemicals, Ltd, Darmstadt, Germany) for 7 days. Cells were split with TrypLE Express and plated adding 10 µM ROCK inhibitor, Y-27632 (Merck Chemicals, Ltd) to enhance cell survival for the initial passages. Compound E was withdrawn after seven days in culture and cells were maintained in the same medium until neuronal differentiation.

Once the allele-specific siRNAs were validated in HEK293T cells, we selected the most efficient siRNA (siM9) to test in NSCs. Pepmute transfection reagent (SignaGen laboratories, Gaithersburg, USA) was used for transfecting NSCs following manufacturer's instructions. We used 50 nM of allele-specific siM9, 5 nM siTDP or 5 nM of sic and analysed cells by immunofluorescence and/or western blot.

### Western blot and densitometry analyses

Cell lysates were obtained by lysing cells with cold Radio-Immunoprecipitation Assay (RIPA) buffer (150 mM sodium chloride, 1.0% NP-40 or Triton X-100, 0.5% sodium deoxycholate, 0.1% SDS (sodium dodecyl sulphate), 50 mM Tris, pH 8.0), incubating on ice for 20 minutes and centrifuging at 14 000 rpm for 30 minutes. Supernatants were mixed with 2× SDS buffer (20% glycerol, 4% SDS, 100 mM Tris pH 6.8, 0.002% Bromophenol blue, 100 mM dithiothreitol) and boiled for 10 minutes, constituting the soluble fraction. Protein concentration was determined by Bio-Rad DC Protein Assay (Hemel Hempstead, UK).

Approximately 10 µg of homogenates were loaded into pre-cast gels (NuPAGE Novex 10% Bis Tris, Invitrogen) and transferred to nitrocellulose membrane using iBlot (Invitrogen). The membranes were blocked with 5% non-fat dry milk for at least one hour at room temperature and incubated with primary antibody overnight at 4°C. Membranes were washed with 1% Tween-20 TBS (TBST) and incubated with fluorescent secondary antibody (Thermo Fisher Scientific Inc, Waltham, MA, USA) in 0.01% SDS-TBS buffer for one hour at room temperature protected from light. After serial washes with TBS buffer, membranes were scanned using the Odyssey Imaging System (Li-Cor Biosciences, Cambridge, UK). Membranes were scanned, avoiding saturation of the bands, and quantified using ImageJ (version 1.45e, NIH, Bethesda, USA, http://rsb.info.nih.gov/ij/). Densitometry and statistical analyses were performed using One-way ANOVA with Bonferroni post-hoc test or Student's t-test using GraphPad Prism 5.03 (GraphPad Software, San Diego, USA). All antibodies were purchased from Sigma-Aldrich unless stated otherwise (Dorset, UK). The antibodies used in western blot were: polyclonal anti-TDP-43 antibody (1∶5 000, ProteinTech Group, Chicago, USA), monoclonal anti HA (1∶10 000), monoclonal anti GFP (1∶1 000), monoclonal anti GAPDH (1∶5 000) and polyclonal anti-histone 3 as a loading control marker (1∶20 000).

### Immunofluorescence

After transfection with siRNAs, NSCs were rinsed with PBS and fixed with 4% paraformaldehyde for 15 minutes at room temperature. Cells were permeabilized with 0.25% Triton-X 100 for 15 minutes at room temperature and blocked with 10% normal donkey serum for one hour. Cells were incubated with primary antibody diluted in 5% normal donkey serum at 4°C overnight. After serial washes with PBS, cells were incubated with secondary antibody for one hour at room temperature, rinsed with PBS and stained with 1.25 µg/ml DAPI (Sigma) for 1–2 minutes at room temperature. Coverslips were mounted using DAKO mounting medium (Dako, Glostrup, Denmark) onto fluorescent microscope slides (Fisher Scientific, Waltham, MA, USA).

Antibodies used for immunostaining were: polyclonal HA (1∶100, Cell Signaling Technology); polyclonal TDP-43 (1∶300, ProteinTech Group); monoclonal TDP-43 (1∶300, Santa Cruz Biotechnology, Inc, Santa Cruz, USA); polyclonal nestin (1∶400, Santa Cruz Biotechnology, Inc) and monoclonal β-III tubulin (1∶400, Sigma-Aldrich). Secondary antibodies were purchased from Jackson ImmunoResearch Laboratories Inc. (West Grove, PA, USA). Cell images were acquired using a Zeiss Axiovert S100 (HB0100) (Carl Zeiss Ltd., Hertfordshire, UK) inverted microscope, a Zeiss LSM 510 META confocal laser scanning microscope and an InCell Analyser 1000 (for aggregation analysis, described below).

### HEK293T aggregation analysis

HA-TDP-43 wt stable transfected HEK293T cells were transiently transfected with C-terminal GFP-TDP-43^wt^ or GFP-TDP-43^M337V^ and siRNAs for 48 hours, fixed and processed for immunofluorescence as described. Images were acquired using an InCell Analyser 1 000 (GE Healthcare, Little Chalfont, UK). Quantification was carried out by counting an average of 315 cells per image, in 25 images per treatment condition (5 sites each from 5 wells of a 96-well plate), for three independent experiments. Cytoplasmic aggregates were counted using the previously validated [33] Cell scoring module within the MetaMorph Image System 7.5 (v. 7.7, Molecular Devices, Wokingham, UK), and data were analysed using GraphPad Prism 5.03.

### Total, nuclear and cytosolic TDP-43 quantification

TDP-43 and DAPI images of NSCs were acquired using identical parameters across all cell lines and treatments, and analysed with MetaMorph Image System. Quantification was carried out by counting an average of 150 cells per image, in 7–10 images for each treatment condition, for three independent experiments. Quantification of total and cytoplasmic TDP-43 was performed using journals written in-house (available from ELS). These measured the staining which was above a user-defined threshold, in the original image set (total TDP-43) and in an image set where a binarized mask of the DAPI-stained nuclear image was subtracted from the TDP-43 image (cytoplasmic TDP-43). Nuclear TDP staining was measured using the Count Nuclei module with TDP-43-stained images. Values shown are the average integrated intensities of staining per cell, with cell counts performed using the Count Nuclei module with DAPI-stained images.

### RNA extraction and cDNA Synthesis

RNA was extracted from cells using the RNeasy Mini Kit (Qiagen) following the manufacturer's instructions followed by DNase digestion. RNA was quantified on a microvolume spectrophotometer (NanoDrop 2000, ThermoScientific) and the quality and integrity was checked using a 1% agarose gel. Only samples with an absorbance ratio at OD260/280 between 1.8 and 2.2, and OD260/230 at about 2.0 with clear 28S/18S bands and no smears were processed for cDNA synthesis.

For cDNA synthesis, 150 ng of total RNA was transcribed with the SuperScript III First-Strand Synthesis System (Life Technologies) in a 20 µL volume, following the manufacturer's instructions. cDNAs were diluted to 12.5 ng/µL and stored at −20°C.

### Transcription analysis by Real-time PCR

The real-time PCRs were performed on a MicroAmp Optical 384-well reaction plate with barcode covered with MicroAmp Optical Adhesive Film (Applied Biosystems). Reactions were manually assembled and contained 0.5 µL of Taqman Gene Expression Assay (Hs00606522, Applied Biosystems), 5 µL of Taqman Gene Expression Master Mix (Applied Biosystems), 2 µL of diluted cDNA and 2.5 µL of nuclease-free water (Ambion). Concurrently, housekeeping endogenous control glyceraldehyde-3-phosphate dehydrogenase (GAPDH) was assayed for each individual sample for normalisation purposes. The PCR profile was: 2 minutes at 50°C, 10 minutes at 95°C, followed by 40 cycles of 15 seconds at 95°C and 1 minute at 60°C. Negative controls (no template cDNA and blank) were included in all assays. RT-PCRs were performed with two biological replicates and three technical replicates of each cDNA sample in the ABI7900HT sequence detection system. On completion of RT-PCR, Ct values were generated using SDS 2.3 software.

The relative level of expression (RQ) for *TARDBP* was calculated based on the formula RQ = 2^−ΔCt^. Each Ct value represents the mean of three values were discarded if they were not within 0.25 standard deviation of the mean. ΔDCt equals to Ct_TARDBP_ – Ct_GAPDH_. The relative level of expression of each sample was normalised to the ΔCt of control NSCs treated with sic.

## Results and Discussion

In recent years, the identification of TDP-43 as a major component of cytoplasmic, detergent-resistant inclusions in ALS and FTLD has opened new areas of research aiming to understand the pathological mechanisms of these diseases.

In order to investigate if an allele-specific siRNA can reduce the expression of TDP-43 mutant allele as a form of therapy; we designed five allele-specific siRNAs containing 19 nucleotides, targeting the M337V mutation. Early studies on the RNAi pathway showed that contiguity at the centre of siRNA is crucial to target recognition and cleavage, since mismatch abolished the RNAi effect and tended to occur at the centre of the siRNA structure [34], [35]. Therefore, the position of the altered nucleotide in the siRNA is important to discriminate the mutant from the wild-type alleles. Allele-specific siRNAs targeting different regions of the same transcript display differences in the efficiency of silencing, therefore we designed siRNAs where the mutated nucleotide G position varies within the siRNA structure (Fig. 1A). The allele-specific siRNAs-M9, M3 and M17 are perfectly matched with the mutant allele but have a single mismatch with the wild-type counterpart. However, it is likely that not all single mismatches would create silencing selectivity and some mismatches might be tolerated by the RISC complex; compromising the allele-specificity. For this reason, we designed siRNAs with more than one mismatch with the wild-type allele. The siRNA-M8-9 contains a double mismatch with the wild-type at positions 8 (sense strand G>C) and at position 9 (the mutation site), hence creating a single mismatch with the mutant allele. Similarly, the siRNA-M5U contains five mismatches (sense strand U>G) with the wild-type and four mismatches with the mutant allele (Fig. 1A).

To determine if these allele-specific siRNAs specifically knock down the M337V allele, HEK293T cells were transiently transfected with full length GFP-TDP-43^wt^ or GFP-TDP-43^M337V^ in combination with allele-specific siRNAs and analysed by western blot. In order to exclude the possibility that co-transfection with siRNA reduced the transfection efficiency of GFP-TDP-43^M337V^ we co-transfected a flag-tagged protein (Fig. 1B). Two siRNAs showed a significant reduction in GFP-TDP-43^M337V^ protein levels (siM9 and siM17). Using the siRNA with mismatch at position 9 (siM9), GFP-TDP-43^M337V^ was reduced by 78%, whereas the allele-specific RNA with a mismatch at position 17 silenced GFP-TDP-43^M337V^ by 25.6%. In contrast, GFP-TDP-43^wt^ levels remained unchanged by transfection with any of the allele-specific siRNAs. Non-targeting siRNA control (sic) and siRNA targeting the N-terminal region of TDP-43 (siTDP-43) were used as controls. As expected, siTDP-43 was able to silence both GFP-TDP-43^wt^ and GFP-TDP-43^M337V^, decreasing their expression levels by approximately 50% (Fig. 1C).

**Figure 2:**
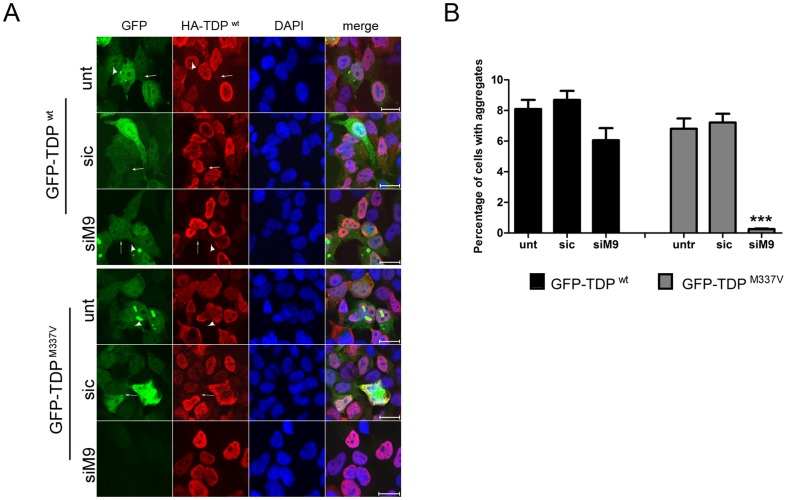
Allele-specific siRNA silences the mutant allele specifically and reduces cytoplasmic inclusions in HEK293 cells. **A.** HA-TDP-43^wt^ stably expressing HEK293 cells were co-transfected with C-terminal GFP-TDP-43^wt^ or GFP-TDP-43^M337V^ and siRNAs for 48 hours. Cells were fixed and stained with HA antibody (red) and DAPI (blue). GFP-TDP-43 cytosolic and nuclear inclusions of different sizes were seen. C-terminal GFP-TDP-43^wt^ and GFP-TDP43^M337V^ inclusions co-localized with full length HA-TDP-43^wt^ (arrows), however some inclusions did not recruit full length HA-TDP-43^wt^ (arrowhead). Scale bars = 20 µm. **B.** Percentage of cells with GFP aggregates. siM9 reduced the number of cells with aggregates in GFP-TDP-43^M337V^ – expressing cells. More than 70 000 cells were counted from three independent experiments. Error bars represent SEM (Student's T-test,*** P<0.001).

Having determined that allele-specific siRNA can reduce protein expression from the mutant allele specifically, we sought to determine whether this RNAi approach could also reduce the formation of cytosolic TDP-43 aggregates. HEK293T cells transiently transfected to overexpress C-terminal fragments of either GFP-TDP-43^wt^ or GFP-TDP-43^M337V^ form cytosolic and occasionally nuclear aggregates (Figure 2A). In contrast, HEK293 stable cell lines expressing full-length HA-TDP-43^wt^ at near physiological levels do not form aggregates. Therefore, by transiently transfecting C-terminal GFP-TDP-43 proteins into HA-TDP-43 expressing cells, we were able to simultaneously assess whether allele-specific knockdown altered the formation of TDP-43^M337V^ aggregates, and whether TDP-43 expression from the other ‘allele’ was maintained.

We observed that after allele-specific siRNA transfection, GFP-TDP-43^M337V^ levels were reduced with a concomitant decrease in detectable cytoplasmic inclusions (Fig. 2B). In addition, we observed that HA-TDP-43^wt^ remained unchanged, suggesting that the allele-specific siM9 specifically reduces the mutant allele (Figure 2A). The majority of the cytoplasmic inclusions of C-terminal GFP-TDP showed recruitment of full length HA-TDP43^wt^ (Figure 2A, arrows); however some cellular aggregates did not (arrowhead), suggesting the formation of different types of cellular aggregates. Cells co-transfected with C-terminal GFP-TDP-43^wt^ and allele-specific siM9 did not show a statistical significant reduction of cellular aggregates.

The HEK293T experiments showed that allele-specific RNAi can selectively reduce GFP-TDP-43^M337V^, with concomitant reduction in the formation of cytoplasmic aggregates.

**Figure 3:**
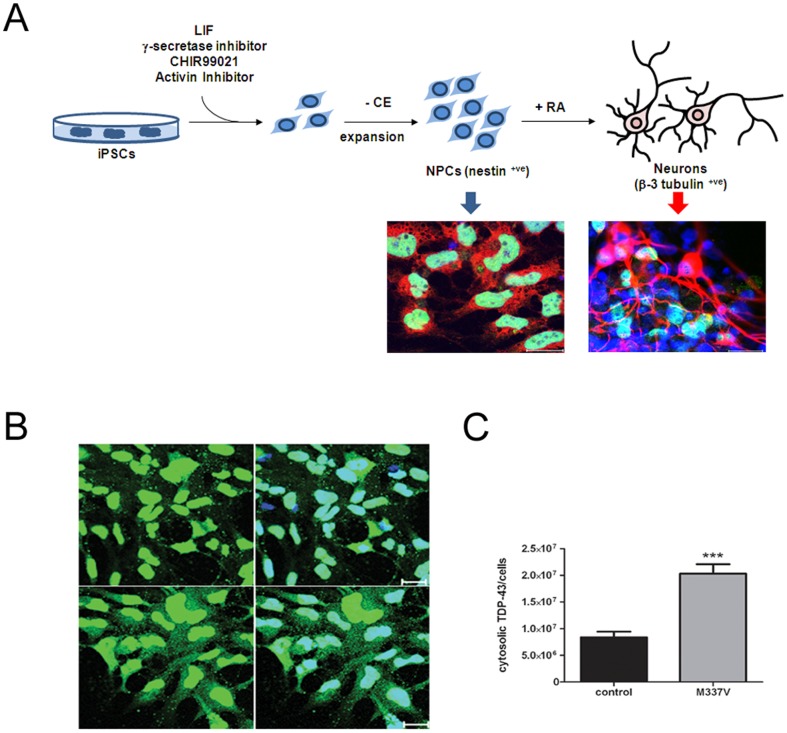
Generation of neural stem cells from iPSC lines. **A.** iPSCs are differentiated into neural stem cells (NSCs) using small molecules for 10 days. At this stage these cells are nestin positive (neuroprecursor marker) and can be further differentiated into neurons after treatment with 1 µM of retinoic acid for 14 days. Neurons are positive for β-III tubulin, which is a neuronal marker. Scale bars = 20 µm. **B.** iPSCs from an ALS patient with M337V mutation and control were differentiated into NSCs and immunostained with TDP-43 antibody. A representative confocal image of TDP-43 immunostaining shows an increase of cytoplasmic TDP-43 in M337V NPCs. **C.** Densitometry quantification of cytoplasmic TDP-43 normalised to the control (n = 3 independent experiments. *** P<0.001).

To test whether allele-specific siRNA could decrease endogenous TDP-43^M337V^ levels in cells derived from patients, we differentiated induced pluripotent stem cells (iPSCs) into neural stem cells (NSCs) (Figure 3). NSCs are transfectable and express the early neural stem cell marker nestin. Under stimulation with retinoic acid, these cells can be differentiated into neurons, which express the neuronal marker β-III tubulin (Fig. 3 A).

NSCs were stained with polyclonal TDP-43 antibody and DAPI and were analysed by quantitative immunofluorescence using Metamorph software (Figure 3B). Approximately 4 500 cells each were counted for the M337V and control lines. The mutant M337V lines showed approximately a two-fold increase in cytoplasmic TDP-43 compared to the controls. Given the importance of subcellular localisation to TDP-43 toxicity, we decided to use an immunofluorescence assay to assess the impact of allele-specific knockdown on nuclear and cytoplasmic TDP-43 levels in NSCs.

**Figure 4:**
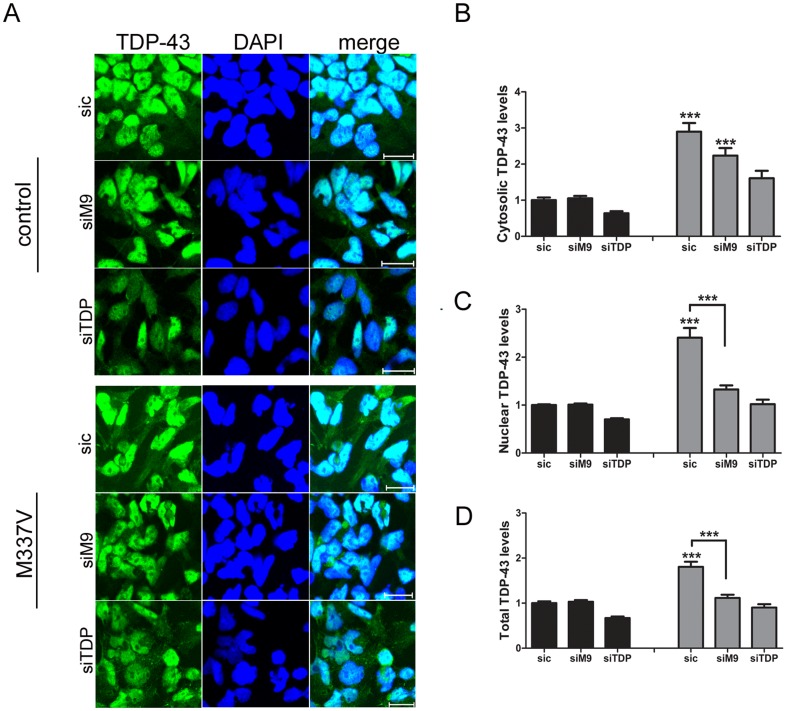
Allele-specific knockdown of M337V allele on neural stem cells. **A.** NSCs were transfected with allele-specific siM9 and stained for TDP-43. Images were acquired using identical parameters and analysed using Metamorph software. Representative confocal immunolabeling images showing allele-specific M337V knockdown in M337V lines. The allele-specific siM9 reduces endogenous TDP-43^M337V^ expression in all compartments (cytosolic (**B**), nuclear (**C**) and total (**D**) TDP-43) (n = 3 independent experiments. ** P<0.01 and *** P<0.001).

**Figure 5:**
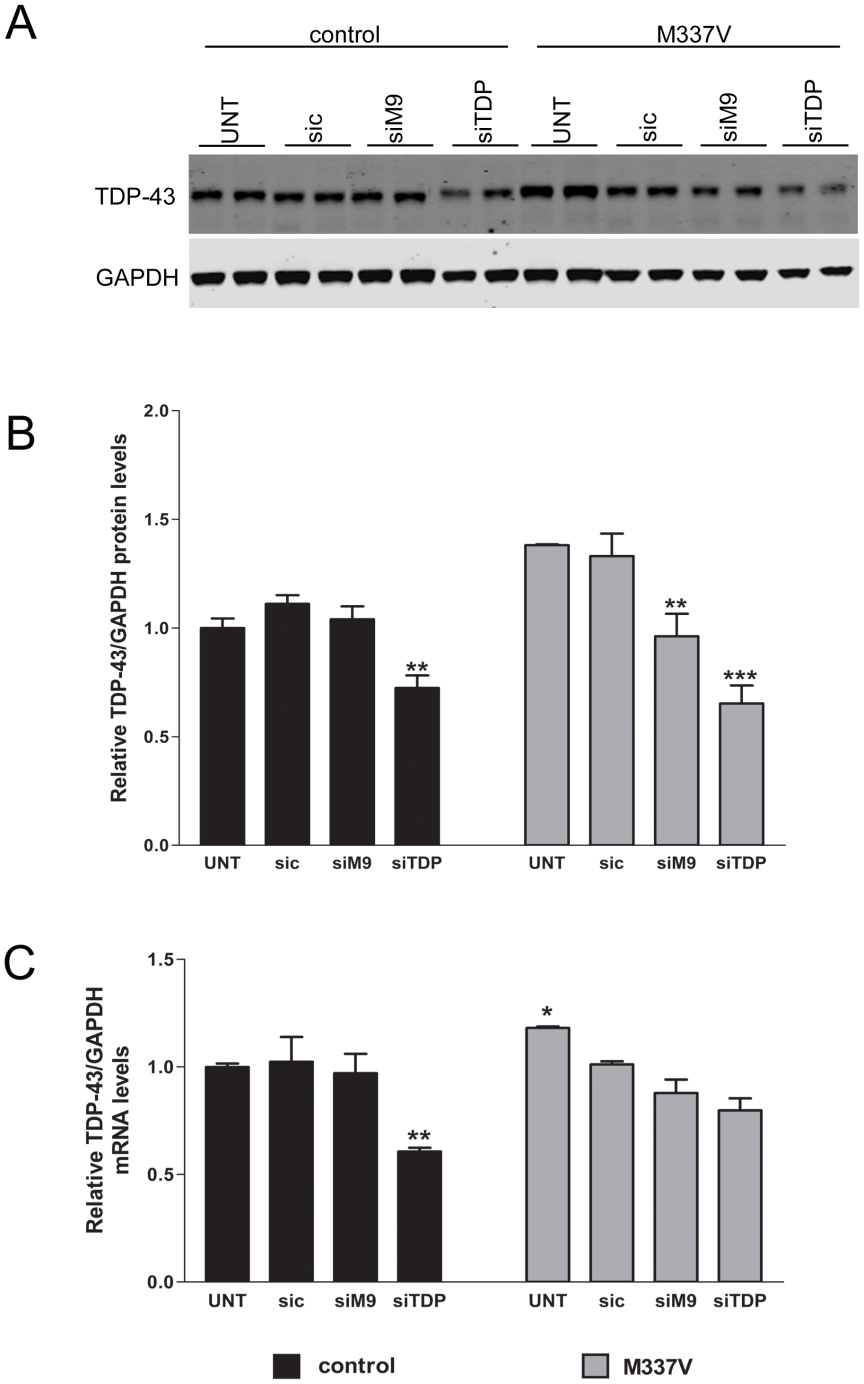
Allele-specific siM9 decreases total TDP-43 transcripts and protein levels in neuralised cells. **A.** Representative Western blot image showing M337V knockdown in M337V lines. **B.** Densitometry analysis of relative TDP-43 protein normalised to GAPDH. **C.** qPCR display unchanged levels of total TDP-43 in the control lines transfected with siM9, whereas M337V lines showed a reduction. Error bars represent SEM (One way ANOVA, * P<0.05, ** P<0.01, *** P<0.001).

We transfected the control and M337V lines with sic, siTDP-43 and allele-specific siM9 and quantified TDP-43 levels in the nuclear or cytoplasmic compartment. As expected, in both control and M337V NSCs, TDP-43 levels (total, nuclear and cytoplasmic) were unchanged when transfected with sic and reduced when transfected with siTDP-43 (P<0.001). NSC ^M337V^ transfected with the allele-specific siM9 showed a reduction of approximately 40% in total TDP-43 (cytosolic+nuclear); 30% in cytosolic TDP-43 and 45% in nuclear TDP-43 compared to controls (Fig. 4A–D). Similarly, qPCR and western blot analyses revealed a decrease in the total levels of the endogenous TDP-43^M337V^, but not TDP-43^wt^, when cells were transfected with allele-specific siRNA (Fig. 5A–C).

TDP-43 has the ability to autoregulate its own protein levels. TDP-43 protein binds to the 3′ untranslated region (UTR) of TDP-43 mRNA and promotes mRNA instability leading to reduced de novo TDP-43 protein synthesis, which, in turn, leads to diminished inhibition and increase in de novo synthesis [5], [36]. Indeed, heterozygous null *TARDBP* mouse models express normal levels of TDP-43, indicating tightly controlled compensation following loss of one allele [37], [38], [39]. Here, however, we demonstrate that knockdown of mutant transcript (siM9) results in an overall decrease in TDP-43 protein levels. Similarly, knockdown of wild-type TDP-43 (siTDP) also reduces TDP-43 protein levels. These findings suggest that there are limits to the extent to which autoregulation can compensate for a loss of transcript, especially following RNA interference. Furthermore, TDP-43 protein levels after allele-specific knockdown approach basal levels in control cell lines, suggesting this might represent a physiological “set-point”, perhaps reflecting full ribosome occupancy, ribosome density, or translation rate of remaining wild-type transcripts. This is a promising finding, which supports the siRNA approach to mitigating TDP-43 overexpression associated with patient mutation.

Together, these studies provide a proof of concept that allele-specific siRNA can specifically silence mutant TDP-43^M337V^, resulting in a decrease of both the full-length as well as C-terminal fragments of mutant TDP-43 in mammalian cell lines. We have also shown for the first time that an allele-specific siRNA can reduce the expression of the endogenous mutant allele in neuralised cells derived from iPSCs from a patient carrying the M337V mutation.

RNA interference is a potential therapeutic tool to treat autosomal dominant diseases; however we still have to learn what the effects of chosen RNAis are in patients with TDP-43 mutation. The knowledge that allele-specific RNA interference specifically decreases expression of mutant TDP-43^M337V^ has a great implication for potential treatment of familial ALS.
